# Face-to-Face Versus Video Assessment of Facial Paralysis: Implications for Telemedicine

**DOI:** 10.2196/11109

**Published:** 2019-04-12

**Authors:** Jian Rong Tan, Susan Coulson, Melanie Keep

**Affiliations:** 1 Discipline of Physiotherapy Faculty of Health Sciences University of Sydney Lidcombe Australia; 2 Faculty of Health Sciences University of Sydney Lidcombe Australia

**Keywords:** telemedicine, eHealth, facial paralysis, facial nerve, outcome assessment (health care), video recording, facial expression, smiling, quality of life, Bell palsy

## Abstract

**Background:**

Patients with facial nerve paralysis (FNP) experience challenges in accessing health care that could potentially be overcome by telemedicine. However, the reliability of telemedicine has yet to be established in this field.

**Objective:**

This study aimed to investigate the consistency between face-to-face and video assessments of patients with FNP by experienced clinicians.

**Methods:**

A repeated-measures design was used. A total of 7 clinicians assessed the FNP of 28 patients in a face-to-face clinic using standardized grading systems (the House-Brackmann, Sydney, and Sunnybrook facial grading systems). After 3 months, the same grading systems were used to assess facial palsy in video recordings of the same patients.

**Results:**

The House-Brackmann system in video assessment had excellent reliability and agreement (intraclass correlation coefficient [ICC]=0.780; principal component analysis [PCA]=87.5%), similar to face-to-face assessment (ICC=0.686; PCA=79.2%). Reliability of the Sydney system was good to excellent, with excellent agreement face-to-face (ICC=0.633 to 0.834; PCA=81.0%-95.2%). However, video assessment of the cervical branch and synkinesis had fair reliability and good agreement (ICC=0.437 to 0.597; PCA=71.4%), whereas that of other branches had good to excellent reliability and excellent agreement (ICC=0.625 to 0.862; PCA=85.7%-100.0%). Reliability of the Sunnybrook system was poor to fair for resting symmetry (ICC=0.195 to 0.498; PCA=91.3%-100.0%) and synkinesis (ICC=−0.037 to 0.637; PCA=69.6%-87.0%) but was good to excellent for voluntary movement (ICC=0.601 to 0.906; PCA=56.5%-91.3%) in face-to-face and video assessments. Bland-Altman plots indicated normal limits of agreement within ±1 between face-to-face and video-assessed scores only for the temporal and buccal branches of the Sydney system and for resting symmetry in the Sunnybrook system.

**Conclusions:**

Video assessment of FNP with the House-Brackmann and Sunnybrook systems was as reliable as face-to-face but with insufficient agreement, especially in the assessment of synkinesis. However, video assessment does not account for the impact of real-time interactions that occur during tele-assessment sessions.

## Introduction

### Background

Facial nerve paralysis (FNP) occurs when there is damage to the seventh cranial nerve, the facial nerve. This affects an individual’s ability to express emotions via facial movements (eg, frowning, smiling, or pouting), thereby compromising interpersonal communication [1-4]. The effects of FNP are not only limited to physical and aesthetic deficits caused by weakness, synkinesis (involuntary muscle movements accompanying voluntary movements), and asymmetry of the resting and moving face [1-3,5-10]. FNP is also associated with functional effects such as oral incontinence, speech deficits, dry eye, and subsequent corneal damage and psychosocial effects such as decreased self-esteem, psychological distress, depression, and reduced quality of life [1-3,5-10]. It is, therefore, desirable that individuals with FNP receive appropriate help for their condition to reduce deficits and improve functional outcomes.

Management of facial nerve disorders is a highly specialized field with a limited number and availability of practitioners. For example, in Australia, there is only 1 major facial nerve center—the Sydney Facial Nerve Clinic (SFNC). In addition to limited access to relevant health professionals, individuals with FNP face common challenges to health care access, which include the direct and indirect cost of care, distance, transportation, mobility, and time constraints [11-15].

One potential way of improving access to care for patients who experience FNP is telemedicine. Telemedicine is health care delivered via telecommunications technology over a distance [11,15-18], allowing improved access to care in various physical and mental health conditions by overcoming geographical and temporal barriers [11,13-21]. Telemedicine has also been associated with increased access and compliance to pulmonary rehabilitation [22] and reduced hospitalization rates, emergency department visits, and length of hospital stay for patients with chronic obstructive pulmonary disease [23]. Patients have reported similar or better quality of life compared with usual care [23]. In addition, internet-based interventions have been found to significantly decrease pain intensity, depression, anxiety, and stress and increase quality of life compared with in-person psychotherapy in patients with medically unexplained pain [20], in youth with depression and anxiety [21], in military personnel with post-traumatic stress disorder and depression [17,24], and in patients with neurofibromatosis [15].

Limitations of telemedicine include technical difficulties such as poor video resolution, connectivity, and sound issues [17,18,20,21,25]. These could potentially compromise the quality of the service provided and are commonly cited reasons for not implementing telehealth [17,18,20,21,25].

Although telemedicine has not yet been formally studied in FNP, it has, anecdotally, been implemented by some clinicians to provide services for patients experiencing geographical barriers to treatment. The above evidence suggests that telemedicine may play a role in increasing access to medical care for patients with FNP. As such, studies are required to establish the reliability of telemedicine as compared with face-to-face health care of FNP.

Tele-assessment of facial function using the appropriate facial grading systems is an integral part of the management of FNP as FNP management requires initial and ongoing assessment of patients’ facial function. The clinician’s overall impression of the patient is taken as standard and is assumed to represent the degree of abnormality of facial function [1,2,4-6,8,26-36]. This enables clinicians to keep track of patients’ recovery and evaluate the efficacy of treatment [4,28,31,37]. FNP is assessed using grading scales that typically measure patients’ facial function, grade symmetry of the face at rest, and measure displacement of facial features during voluntary movement as well as in the presence of synkinesis. Previous research has shown that greatest displacement during facial expressions occurs in the vertical axis of the frontal plane, followed by the anterior-posterior axis of the sagittal plane and the horizontal axis of the frontal plane [27]. This raises the question of whether tele-assessment of the patient, which would present the patient in a 2-dimensional (2D) view, would be as reliable and valid as a face-to-face assessment where patients are presented in a 3-dimensional (3D) view.

### Objectives

Despite the significant body of literature on the applications of telemedicine, there has been a lack of research conducted on its use for the management of FNP. This exploratory study aims to compare the reliability of 3 commonly used FNP grading systems when administered via static video and face-to-face. Face-to-face assessment presents the patient in a 3D view, whereas the frontal video shows the patient in a 2D view that would not take into account the anterior-posterior axis of the sagittal plane. Therefore, it was hypothesized that reliability across the 3 grading systems would differ between the face-to-face and video assessments in movements where there was significant anterior-posterior axis displacement, for example, the pout, smile, and snarl.

## Methods

### Study Design

A repeated-measures design was undertaken in which the face-to-face and video assessments were conducted on the same patients and assessed by the same assessors. The video assessment was performed on a static prerecorded video of the patients taken before the face-to-face assessment. There was a 3-month delay between the face-to-face and video assessments to minimize retention of gradings used by the assessors. The 3-month period was based on a similar study by Banks et al [31], which compared in-person and video assessment of facial mimetic function using the newly developed eFACE facial grading system.

### Participants

Participants comprised clients at the SFNC who presented with a diagnosed FNP between August 2016 and March 2017. Assessment was conducted in a standard, routine manner, and the video assessment segment of the study did not require additional involvement by the participants, nor did it preclude them from receiving treatment or other appropriate interventions. Ethical approval was granted by the Ethics Review Committee (RPAH Zone) of the Sydney Local Health District (Protocol No X17-0013 ERC).

### Procedure

The study took place between August 2016 and March 2017. Patients of the SFNC are routinely video-recorded while performing a protocol of facial movements before receiving treatment. Videos were recorded in the frontal view and included movements such as eyebrow raise, eye closure, smile, snarl, and pucker. This is not a recording of a live videoconference. Static videos were used as it allowed researchers to control for individual differences in how clinicians guided patients through assessment protocols in a live videoconferencing session. In addition, it was important that the patients’ state of facial paralysis at the face-to-face and video assessments were identical. Some recovery of facial function might have occurred if the videoconferencing assessment was conducted after the face-to-face assessment.

After the recording is taken, participants commence their in-person clinic session where the face-to-face assessment was conducted. Moreover, 3 months later, each assessor rated the video footage taken at the initial face-to-face session. The order in which the videos were shown was randomized to control for rater fatigue. Each assessor graded each patient face-to-face with the House-Brackmann system, the Sunnybrook system, and the Sydney system and then again in the video assessment.

Face-to-face assessment was the standard against which the video assessment was compared. This process enabled inferences to be made about tele-assessment as a potential substitute for face-to-face assessment.

### House-Brackmann Facial Grading System

The House-Brackmann facial grading system is the most commonly used system to measure FNP [2-5,8,26-28,30, 31,33,34,37]. It is a gross scale ranked from 1 to 6, with each grade giving an overall impression of facial function, resting symmetry, and synkinesis [4,5,28,34]. However, the House-Brackmann system is limited in that it does not allow for regional assessment, the range of scores does not reflect the clinically important change, and lacks sufficient classification of synkinesis [8,26,30,32,34-36]. This has led to the administration of a range of alternative systems being used by practitioners around the world.

### Sydney Facial Grading System

The Sydney Facial Grading System, which assesses voluntary movement of the 5 branches of the facial nerve and overall synkinesis, has also been used regionally in Australia [1,6,26] and reported in the International Facial Nerve Symposium conference proceedings over a 20-year period.

### Sunnybrook Facial Grading System

The Sunnybrook facial grading system is a regional weighted system that assesses resting symmetry, voluntary movements, and synkinesis of the face, after which a composite score on a 100-point scale is computed [28,34]. It grades patients in a more objective and continuous manner than the House-Brackmann system, and each component of the system is sensitive to change and contributes to a change in the composite score [1,3,8,26,28,30,32,34,35].

### Analyses

Participants’ data were deidentified before analyses. Quantitative analyses were conducted using the Statistical Package for the Social Sciences. Descriptive statistics were used to summarize demographic information. A significance level of *P*<.05 and corresponding 95% CIs were used for all inferential statistics.

Agreement between face-to-face and video House-Brackmann grades, Sydney scores, and Sunnybrook scores was assessed using the 1-way random, single measure intraclass correlation coefficient (ICC, 1,1), and 2-tailed repeat measures *t* tests. ICC (1,1) was selected because each participant was assessed by a different set of assessors. For each participant, scores given by 3 randomly selected assessors were used to compute the ICC; hence, the rater was a random effect [38,39]. Portney and Watkins [38] noted that “ICC ranges between 0.00 and 1.00, with values closer to 1.00 represent stronger reliability,” but there were “no standard values for acceptable reliability using the ICC,” and that the researcher should determine the level of reliability needed to justify the use of the tool being assessed. On the basis of the study by Banks et al [31], we defined reliability as poor for ICC values less than 0.40, fair for values between 0.40 and 0.59, good for values between 0.60 and 0.74, and excellent for values 0.75 and above. The 95% CI indicates that there is a 95% chance that any score would lie within the range [39].

As the ICC could be affected by outlying data, the percentage exact agreement (PEA) and percentage close agreement (PCA) were calculated to make up for the potential deficiency [40]. PEA is the proportion of cases where all 3 assessors gave the same grade for each participant. PCA is similar to PEA but includes cases where there was a maximum difference of 1 grade between assessors [40]. Agreement of PEA with ICC suggests the data are sound [40]. However, as a 1-grade and 17-point difference between assessors had been determined to be reasonable for the grading systems and the Sunnybrook composite score, respectively [4,32], we will be comparing the ICC with the PCA instead. Similar to the ICC, agreement was defined as poor for PCA values less than 40%, fair for values between 40% and 59%, good for values between 60% and 74%, and excellent for PCA values 75% and above.

Repeat measures *t* tests were administered on a per-protocol analysis. Missing data (ie, no matched pairs of face-to-face and video assessment grades) were discarded.

Bland-Altman plots were constructed where differences between face-to-face and video assessments were plotted against the means of the 2 assessment modes to determine agreement between the two and whether video assessment can substitute face-to-face assessment with an acceptable degree of error [38,41,42]. Horizontal lines were drawn at the mean difference and at the upper and lower limits of agreement, which are defined as the mean difference plus and minus 1.96 times the SD of the differences, respectively [38,41,42]. Assuming normal distribution, between the limits of agreement is an agreement interval within which 95% of the differences of video assessment fall, compared with face-to-face assessment [38,43]. The Bland-Altman method only defines the intervals of agreement but not whether those limits are acceptable [43]. We have, therefore, defined a difference of 1 grade to be acceptable between House-Brackmann grades and components of the Sydney and Sunnybrook systems and a difference of 17 points in the Sunnybrook composite score.

## Results

### Participants

A total of 28 participants and 7 assessors took part in the study. The mean (SD) age of the 28 patient participants (12 males and 16 females) was 41.7 (15.4) years, with a mean (SD) of 6.5 (6.9) years since FNP onset. There has been no prior research comparing face-to-face and video assessments of FNP, so the sample size (n=28) was based on a related study with 21 participants where there was sufficient power to detect differences between the House-Brackmann, Sydney, and Sunnybrook systems [26]. The etiologies of the FNP are presented in Table 1.

The assessors comprised ear, nose, and throat specialists; head and neck surgeons; and plastic surgeons who regularly attended the SFNC and had at least 10 years of experience in managing facial nerve disorders.

### Face-to-Face Versus Video-Assessed House-Brackmann Grades

The House-Brackmann system administered face-to-face had good reliability and excellent agreement, whereas that administered over a video had excellent reliability and agreement (Table 2). Significant between-subject effects are denoted by significant *P* value in an *F* test with true value 0. When the between-subject effect is significant (*P*<.05), participants are then different from each other, which is a necessary condition for reliability testing [38].

The Bland-Altman plot indicated that the mean difference between House-Brackmann grades given in the face-to-face and video assessments was 0.05, but limits of agreement were between −1.79 to +1.89.

### Face-to-Face Versus Video-Assessed Sydney System Scores

Reliability in assessing the 5 branches of the facial nerve face-to-face using the Sydney system was good to excellent, with excellent agreement (Table 3). However, video assessment of the cervical branch and synkinesis had only fair reliability and good agreement, whereas video assessment of the other branches had similarly good to excellent reliability and excellent agreement.

Inspection of the Bland-Altman plots showed that the mean differences between face-to-face and video assessments were close to 0 when assessing all facial nerve branches using the Sydney system. However, limits of agreement between the 2 modes of assessment were only close to ±1 in assessment of the temporal and buccal branches, and a patient could potentially be graded more than 1 grade apart between the face-to-face and video assessments of the zygomatic, marginal mandibular, and cervical branches and synkinesis.

**Table 1:** The etiology of facial nerve paralysis in study participants.

Diagnoses	Statistics, n (%)	Type
Trauma or iatrogenic	13 (46)	Removal of acoustic neuromas, parotid gland tumors, hemangioma, and basal cell carcinoma
Bell’s palsy	5 (18)	Idiopathic
Tumor	4 (14)	Facial nerve neuroma
Herpes zoster oticus	3 (11)	Viral (Ramsay Hunt Syndrome)
Congenital	2 (7)	Developmental
Trauma or accidental	1 (4)	Gunshot wound

**Table 2:** Reliability and agreement of the House-Brackmann scale across assessment modes.

Assessment mode	Intraclass correlation coefficient (95% CI)	*P* value	Percentage exact agreement (%)	Percentage close agreement (%)
Face-to-face	0.686 (0.488-0.835)	<.001	41.7	79.2
Video	0.780 (0.623- 0.889)	<.001	25.0	87.5

**Table 3:** Reliability and agreement of Sydney system facial nerve branches across assessment modes.

Sydney system facial nerve branches	Assessment mode	Intraclass correlation coefficient (95% CI)	*P* value	Percentage exact agreement (%)	Percentage close agreement (%)
Temporal	Face-to-face	0.789 (0.624-0.900)	<.001	52.4	95.2
	Video	0.857 (0.734-0.934)	<.001	66.7	90.5
Zygomatic	Face-to-face	0.633 (0.402-0.813)	<.001	33.3	90.5
	Video	0.625 (0.392-0.809)	<.001	42.9	85.7
Buccal	Face-to-face	0.834 (0.695-0.922)	<.001	61.9	95.2
	Video	0.862 (0.742-0.936)	<.001	61.9	100.0
Marginal mandibular	Face-to-face	0.794 (0.630-0.902)	<.001	52.4	85.7
	Video	0.758 (0.576-0.883)	<.001	33.3	85.7
Cervical	Face-to-face	0.790 (0.624-0.900)	<.001	42.9	81.0
	Video	0.597 (0.356-0.792)	<.001	38.1	71.4
Synkinesis	Face-to-face	0.709 (0.504-0.857)	<.001	57.1	90.5
	Video	0.437 (0.172-0.687)	<.001	42.9	71.4

### Face-to-Face Versus Video-Assessed Sunnybrook Scores

The reliability of face-to-face assessment using the Sunnybrook system was similar to video assessment across most parameters. The reliability was generally poor to fair when assessing resting symmetry and synkinesis but was good to excellent when assessing voluntary movement (Table 4). Agreement was generally good to excellent across parameters for both face-to-face and video assessments. The Sunnybrook composite score had excellent reliability in both face-to-face and video assessments.

The *F* test for between-subjects effect was not significant in the face-to-face assessment of resting symmetry of the eye and in both modes of assessment of synkinesis in forehead wrinkle, indicating no significant difference amongst patients’ resting symmetry of the eye and presence of synkinesis in the forehead wrinkle.

No PCAs were calculated for the weighted total resting symmetry and weighted total voluntary movement scores. As the raw scores were multiplied by 5 and 4, respectively, to determine the weighted total scores, there would not be cases where scores differed by only 1 point. No PEAs and PCAs were calculated as well for the composite score, which was derived from a formula in which resting symmetry, voluntary movement, and synkinesis were weighted differently. A total of 2 patients with similar Sunnybrook composite scores might, therefore, present differently, with 1 patient scoring better in resting symmetry and the other scoring better in voluntary movement or synkinesis, so agreement in scores might not necessarily reflect similar facial function anyway.

The Bland-Altman plots demonstrated mean differences of close to 0 between face-to-face and video assessments of resting symmetry when using the Sunnybrook system. Limits of agreement between the 2 modes of assessment of resting symmetry were overall close to ±1. Assessment of voluntary movement with the Sunnybrook system saw mean differences of close to 0 between face-to-face and video assessments. Limits of agreement between the 2 modes of assessment of voluntary movement were largely more than ±1 and closer to ±2. Assessment of synkinesis with the Sunnybrook system saw mean differences of close to 0 between face-to-face and video assessments. Limits of agreement between the 2 modes of assessments of synkinesis were overall more than ±1 and closer to ±2. The Bland-Altman plots also indicated a mean difference of 1.52 points in the Sunnybrook composite score between face-to-face and video assessments, which was within the previously defined reasonable limits of 17 points but with limits of agreement greater than ±17 points. Missing data were removed and not included in the results.

**Table 4:** Reliability and agreement of Sunnybrook system parameters across assessment modes. No percentage close agreement (PCA) was calculated for the weighted total resting symmetry and weighted total voluntary movement scores because raw scores were multiplied by 5 and 4 respectively to determine the weighted total scores; there would not be cases where scores differed by only 1 point. No PEA and PCAs were calculated for the composite score, which was derived from a formula in which resting symmetry, voluntary movement, and synkinesis were weighted differently.

Sunnybrook system components		Assessment mode	Intraclass correlation coefficient (95% CI)	*P* value	Percentage exact agreement (%)	Percentage close agreement (%)
**Resting symmetry**						
	Eye	Face-to-face	0.195 (−0.045 to 0.480)	.06	43.5	100.0
		Video	0.367 (0.113 to 0.625)	<.001	73.9	100.0
	Cheek	Face-to-face	0.332 (0.078 to 0.597)	.01	43.5	91.3
		Video	0.498 (0.250 to 0.719)	<.001	52.2	95.7
	Mouth	Face-to-face	0.235 (−0.011 to 0.516)	.03	43.5	100.0
		Video	0.314 (0.061 to 0.582)	.01	47.8	100.0
	Total x 5	Face-to-face	0.548 (0.308 to 0.753)	<.001	34.8	—^a^
		Video	0.544 (0.303 to 0.750)	<.001	26.1	—
**Voluntary movement**						
	Forehead wrinkle	Face-to-face	0.859 (0.74 to 0.932)	<.001	43.5	82.6
		Video	0.906 (0.82 to 0.955)	<.001	60.9	87.0
	Eye closure	Face-to-face	0.601 (0.367 to 0.790)	<.001	22.7	77.3
		Video	0.688 (0.481 to 0.842)	<.001	18.2	77.3
	Smile	Face-to-face	0.741 (0.561 to 0.869)	<.001	26.1	78.3
		Video	0.818 (0.677 to 0.911)	<.001	34.8	91.3
	Snarl	Face-to-face	0.707 (0.512 to 0.850)	<.001	21.7	69.6
		Video	0.806 (0.659 to 0.905)	<.001	39.1	82.6
	Lip pucker	Face-to-face	0.690 (0.489 to 0.840)	<.001	34.8	56.5
		Video	0.826 (0.691 to 0.915)	<.001	69.6	82.6
	Total x 4	Face-to-face	0.812 (0.668 to 0.908)	<.001	8.7	—
		Video	0.912 (0.834 to 0.958)	<.001	21.7	—
**Synkinesis**						
	Forehead wrinkle	Face-to-face	−0.006 (−0.203 to 0.276)	.50	52.2	73.9
		Video	−.037 (−0.225 to 0.241)	.60	43.5	69.6
	Eye closure	Face-to-face	0.248 (0.000 to 0.527)	.03	56.5	73.9
		Video	0.299 (0.047 to 0.570)	.01	34.8	73.9
	Smile	Face-to-face	0.562 (0.325 to 0.762)	<.001	52.2	69.6
		Video	0.527 (0.284 to 0.739)	<.001	52.2	78.3
	Snarl	Face-to-face	0.475 (0.225 to 0.703)	<.001	52.2	73.9
		Video	0.564 (0.327 to 0.763)	<.001	43.5	78.3
	Lip pucker	Face-to-face	0.459 (0.208 to 0.692)	<.001	43.5	73.9
		Video	0.637 (0.418 to 0.809)	<.001	39.1	87.0
	Total	Face-to-face	0.534 (0.292 to 0.744)	<.001	34.8	52.2
		Video	0.475 (0.225 to 0.703)	<.001	30.4	39.1
Composite		Face-to-face	0.811 (0.666 to 0.907)	<.001	—	—
		Video	0.845 (0.720 to 0.925)	<.001	—	—

^a^Missing data.

## Discussion

### Principal Findings

Reliability of the House-Brackmann, Sydney, and Sunnybrook systems was largely similar between face-to-face assessment and video assessment of FNP, despite the 3D nature of face-to-face assessment versus the 2D nature of video assessment. There was, however, poor reliability of assessment of synkinesis.

### Face-to-Face Versus Video-Assessed House-Brackmann Grades

Reliability of the House-Brackmann scale was good in the face-to-face assessment and excellent in the video assessment, with excellent agreement within both modes of assessment. The high level of reliability supports the findings of Evans et al [4], House and Brackmann [33], Coulson et al [26], and Kanerva et al [32]. The lack of an anterior-posterior axis in the video assessment did not appear to affect reliability of the House-Brackmann scale negatively, as implied by the higher ICC value than in face-to-face assessment.

However, the Bland-Altman plot indicated that patients could potentially be given more than 1 grade difference between the face-to-face and video assessments. One grade of difference reflects significant variation in facial function; hence, using the House-Brackmann scale in tele-assessment may give the clinician an erroneous impression of the patient’s condition. Therefore, although the reliability of the House-Brackmann scale indicated that its implementation in a video assessment may yield comparable grades to face-to-face assessment, potential large differences between the 2 assessment modes meant that video assessment using the House-Brackmann scale cannot necessarily substitute face-to-face assessment.

### Face-to-Face Versus Video-Assessed Sydney System Scores

Reliability in assessing the 5 branches of the facial nerve face-to-face using the Sydney system was good to excellent, with excellent agreement. Reliability and agreement were largely similar between face-to-face and video assessments, except for video assessment of the cervical branch and synkinesis, which were less reliable and had a lower agreement than the face-to-face assessment. This supports Coulson et al’s [26] findings of good reliability in assessing function of the facial nerve branches, although the ICC values reported were slightly lower, and poor reliability in assessment of synkinesis. The higher ICC values in this study could be attributed to the slightly larger number of participants (n=28 vs n=21) and possibly fewer outliers, which is supported by the excellent agreement. A further explanation for this finding may be that the Sydney system is commonly used by the assessors in this study.

There are a few factors that could have affected reliability of video assessment of the cervical branch. First, the cervical branch of the facial nerve innervates the platysma muscle, which tightens the anterior neck region and contributes to downward movement of the lower lip [44]. Downward movement of the lips has been found to have significantly more anterior-posterior axis than horizontal axis displacement, so the 2D nature of video assessment of the cervical branch could have affected reliability [27]. Furthermore, 2 assessors had noted poor visibility of the neck region during video assessment of patients who were wearing a hijab or jewelry around the neck. As the neck region is where the platysma muscle is situated, poor visibility of the area would have affected video assessment of the cervical branch. In a face-to-face assessment or tele-assessment over real-time videoconference, the assessor would be able to request the patient to remove any obstructions.

Consistently poor reliability for synkinesis assessment was observed between face-to-face and video assessments. As synkinesis is a multidimensional involuntary movement that occurs simultaneously in an area of the face different from the area being examined for voluntary movement (eg, during smiling, there could be synkinesis in the eye squinting) [26], it could be missed by the clinician who is directing attention to the area being examined for voluntary movement, which could happen in both face-to-face and tele-assessments. In video-based tele-assessment though, there is potential to pause and rewind footage to check for synkinesis.

The Bland-Altman plot indicated that patients could potentially be graded within 1 grade apart between face-to-face and video assessments of the temporal and buccal branches but more than 1 grade apart for that of the zygomatic, marginal mandibular, and cervical branches and synkinesis. This was not surprising as these movements (eg, forehead raise, nose wrinkle, smile, eye closure, and movement of the lower lips) produce a significant displacement in the anterior-posterior axis [27], which is difficult to adequately assess during a video assessment in the frontal plane.

In contrast, during a face-to-face assessment or tele-assessment over a videoconference media such as Skype, the clinician would have the opportunity to request a profile view of the patient, which would take into account movement occurring in the anterior-posterior axis. The clinician may also be able to request the removal of any obstruction for better visibility of the area being examined in real-time tele-assessment. Future research can build on current findings and compare the reliability of static video assessment with live videoconferencing assessment of facial paralysis.

### Face-to-Face Versus Video-Assessed Sunnybrook System Scores

When implemented in face-to-face settings, reliability and agreement of the Sunnybrook system were similar to when used over a video. Reliability was poor to fair when assessing resting symmetry and synkinesis but good to excellent when assessing voluntary movement. Overall, agreement was generally good to excellent across all parameters. Similar to this study, previous research [26] has recorded good ICC reliability scores for the assessment of voluntary movement when using the Sunnybrook system; however, poor ICC reliability scores have been found for the assessment of synkinesis.

As synkinesis has been shown to be difficult to measure, clinicians may have asked patients to repeat and vary the speed and intensity of movements in the face-to-face setting to clarify their assessment finding. They, however, may not have chosen to rewind the video assessments when making ratings for this study. Furthermore, clinicians could not request a movement variation in terms of speed or intensity on the video, which may have unmasked the synkinesis as is sometimes done in face-to-face settings.

Second, studies on FNP have found patients’ quality of life and emotional health to be affected to a larger extent by functional deficits caused by asymmetry in voluntary movements, such as the inability to smile symmetrically and oral incontinence, but to a smaller extent by synkinesis and resting symmetry [1,2,9,37]. Hence, assessors might have prioritized the rating of voluntary movement over resting symmetry and synkinesis as a result of their own personal experience in dealing with what patients perceive as their primary impairments. Third, the low ICC and fairly high PEA and PCA were indicators of outliers skewing the reliability measure or a restriction of range in the grades [40].

Again, reliability was similar and poor between face-to-face and video assessments. In addition, Kayhan et al [45] had found substantial reliability while using the Sunnybrook system to assess videos of patients, whereas Banks et al [31] had found excellent reliability when using the eFACE system to rate videos of participants. The poor reliability for synkinesis assessment may, therefore, be attributed to subjectivity in determining the degree of synkinesis among assessors instead of the 2D nature of video assessment.

In this study, the Sunnybrook composite score had excellent reliability in both face-to-face and video assessments. Hu et al [46], Ross and Nedzelski [35], and Kanerva et al [32] reported similar ICCs for the composite score, indicating excellent reliability. However, the components of the Sunnybrook system comprising resting symmetry, voluntary movement, and synkinesis would give more information about a patient’s facial function during management of FNP than the composite score alone.

Bland-Altman plots indicated that video-assessed mean Sunnybrook scores for resting symmetry, voluntary movement, and synkinesis were similar to the face-to-face assessment scores. However, limits of agreement were too large when comparing video assessment of voluntary movement, synkinesis, and composite scores with face-to-face assessment, although they were reasonable for assessment of resting symmetry. This was expected as there is no displacement in the face at rest when no facial expression is performed, so most of what a clinician would be assessing would be in the vertical and horizontal axes of the frontal plane, and the lack of an anterior-posterior axis is not expected to make a difference in video assessment.

Overall, video-assessed Sunnybrook scores were generally as reliable as face-to-face assessed scores, but limits of agreement were too large between video and face-to-face assessments. This suggests that the Sunnybrook system could reasonably be implemented over a video, but video assessment would not necessarily substitute face-to-face assessment until there is further evidence proving reasonable limits of agreement between face-to-face and video assessments.

### Limitations and Future Research

Although this exploratory study presents interesting findings about the potential for computer-mediated assessments of FNP, there are some limitations. First, the use of recorded videos, rather than real-time videoconferencing, may have limited ecological validity. In tele-health settings, assessment is interactive; patients can be asked to repeat or vary movements and to move the camera for closer or different views of particular movements. This is an opportunity for future research, to compare tele-assessment in real time, and to understand the impact of clinician-patient interactions on tele-assessment accuracy and reliability. It would also enable investigation into whether clinicians are likely to ask patients to repeat a movement despite not rewinding prerecorded videos of patients in this study.

Second, synkinesis was consistently poorly assessed as has been found in previous studies. To improve reliability of assessing synkinesis overall, regardless of the grading system used, protocols on assessing synkinesis could be improved and standardized, with additional notes about the need to review for synkinesis in the same area potentially multiple times. There could also be more specific and relevant training provided toward the protocols [26].

Third, the inter-rater reliability of expert assessors using facial nerve grading scales has been previously demonstrated; therefore, it was not evaluated in this study as each participant was evaluated by a different combination of assessors. A future study could potentially investigate this with a larger sample size.

Finally, the videos recorded in this study were of 1080p quality resolution, which is among the highest definition video modes available currently. Quality of the video assessment could potentially hinder reliability of data [22,23,25], which may affect observations, particularly if the changes in movement were subtle rather than very obvious.

### Implications for Tele-Assessment of Facial Nerve Paralysis

This study suggests that further work is required for the 2D flat screen interface used in tele-health to be comparable with the face-to-face assessment of facial nerve disorders. The video assessment of FNP using the House-Brackmann facial grading scale and Sunnybrook facial grading system was generally as reliable as face-to-face assessment, though there was insufficient agreement between video and face-to-face assessments. This study also showed that the differences in reliability between face-to-face and video assessments using the Sydney system could likely be attributed to the lack of an anterior-posterior axis in the video assessment and generally poor reliability in assessment of synkinesis. Video assessment, however, does not take into account the opportunity for real-time clinician-patient interaction that would present in tele-assessment, which would allow clinicians to request patients to turn to their sides to show a profile view, to repeat certain movements, and to remove viewing obstructions such as face and neck coverings and jewelry, thereby potentially improving agreement between face-to-face and tele-assessments in all 3 facial grading systems. As access to specialist care may pose an additional challenge for patients with FNP, future research could also compare the direct and indirect costs of telepractice with face-to-face management of FNP.

### Conclusions

Our findings highlight the need for further research into the use of the House-Brackmann, Sydney, and Sunnybrook systems in tele-assessment of patients’ facial function. Although reliability of face-to-face scores is similar to reliability of video-assessed scores, there is insufficient agreement between assessments in both modalities to strongly recommend the reliability of its use with current protocols, especially when synkinesis is present. This suggests that tele-assessment has future potential; however, research into the effect of fine-tuning facial movement protocols for use over the 2D screen to maximize reliability of a Web-based assessment is recommended. As evidence to support usage grows, the use of tele-health could increase access to specialized services for individuals with FNP, thereby improving quality of care and rehabilitation outcomes [12,18,19,23,25].

## Abbreviations

2D: 2-dimensional
3D: 3-dimensional
FNP: facial nerve paralysis
ICC: intraclass correlation coefficient
PCA: principal component analysis
PEA: percentage exact agreement
SFNC: Sydney Facial Nerve Clinic
